# SARS‐CoV‐2 innate effector associations and viral load in early nasopharyngeal infection

**DOI:** 10.14814/phy2.14761

**Published:** 2021-02-24

**Authors:** Theodore G. Liou, Frederick R. Adler, Barbara C. Cahill, David R. Cox, James E. Cox, Garett J. Grant, Kimberly E. Hanson, Stephen C. Hartsell, Nathan D. Hatton, My N. Helms, Judy L. Jensen, Christiana Kartsonaki, Yanping Li, Daniel T. Leung, James E. Marvin, Elizabeth A. Middleton, Sandra M. Osburn‐Staker, Kristyn A. Packer, Salika M. Shakir, Anne B. Sturrock, Keith D. Tardif, Kristi J. Warren, Lindsey J. Waddoups, Lisa J. Weaver, Elizabeth Zimmerman, Robert Paine

**Affiliations:** ^1^ Division of Respiratory Critical Care and Occupational Pulmonary Medicine Department of Internal Medicine School of Medicine University of Utah Salt Lake City UT USA; ^2^ Center for Quantitative Biology University of Utah Salt Lake City UT USA; ^3^ Department of Mathematics and School of Biological Sciences University of Utah Salt Lake City UT USA; ^4^ Nuffield College Oxford UK; ^5^ Department of Biochemistry School of Medicine University of Utah Salt Lake City UT USA; ^6^ Metabolomics, Proteomics and Mass Spectrometry Core School of Medicine University of Utah Salt Lake City UT USA; ^7^ Division of Infectious Diseases Department of Internal Medicine School of Medicine University of Utah Salt Lake City UT USA; ^8^ Department of Pathology University of Utah Salt Lake City UT USA; ^9^ ARUP Laboratories Salt Lake City UT USA; ^10^ Division of Emergency Medicine Department of Surgery University of Utah Salt Lake City UT USA; ^11^ Clinical Trial Service Unit & Epidemiological Studies Unit and Medical Research Council Population Health Research Unit Nuffield Department of Population Health University of Oxford Oxford UK; ^12^ Flow Cytometry Core Laboratory University of Utah Health Salt Lake City UT USA; ^13^ Department of Veterans Affairs Medical Center Salt Lake City UT USA

**Keywords:** biomarkers, host shut‐off, innate immunity, interferon, SARS‐CoV‐2

## Abstract

COVID‐19 causes severe disease with poor outcomes. We tested the hypothesis that early SARS‐CoV‐2 viral infection disrupts innate immune responses. These changes may be important for understanding subsequent clinical outcomes. We obtained residual nasopharyngeal swab samples from individuals who requested COVID‐19 testing for symptoms at drive‐through COVID‐19 clinical testing sites operated by the University of Utah. We applied multiplex immunoassays, real‐time polymerase chain reaction assays and quantitative proteomics to 20 virus‐positive and 20 virus‐negative samples. *ACE*‐*2* transcripts increased with infection (OR =17.4, 95% CI [CI] =4.78–63.8) and increasing viral N1 protein transcript load (OR =1.16, CI =1.10–1.23). Transcripts for two interferons (IFN) were elevated, *IFN*‐*λ1* (OR =71, CI =7.07–713) and *IFN*‐*λ2* (OR =40.2, CI =3.86–419), and closely associated with viral *N1* transcripts (OR =1.35, CI =1.23–1.49 and OR =1.33 CI =1.20–1.47, respectively). Only transcripts for *IP*‐*10* were increased among systemic inflammatory cytokines that we examined (OR =131, *CI =1*.01–2620). We found widespread discrepancies between transcription and translation. IFN proteins were unchanged or decreased in infected samples (IFN‐γ OR =0.90 CI =0.33–0.79, IFN‐λ2,3 OR =0.60 CI =0.48–0.74) suggesting viral‐induced shut‐off of host antiviral protein responses. However, proteins for *IP*‐*10* (OR =3.74 CI =2.07–6.77) and several interferon‐stimulated genes (ISG) increased with viral load (*BST*‐*1* OR =25.1, CI =3.33–188; *IFIT1* OR =19.5, CI =4.25–89.2; *IFIT3* OR =245, CI =15–4020; *MX*‐*1* OR =3.33, CI =1.44–7.70). Older age was associated with substantial modifications of some effects. Ambulatory symptomatic patients had an innate immune response with SARS‐CoV‐2 infection characterized by elevated IFN, proinflammatory cytokine and ISG transcripts, but there is evidence of a viral‐induced host shut‐off of antiviral responses. Our findings may characterize the disrupted immune landscape common in patients with early disease.

## Graphical abstract

SARS‐CoV‐2 disrupts the innate immune landscape. Interferon‐lambda transcripts are increased but protein production may be blocked by viral shut‐off. However, key interferon‐stimulated antiviral genes are transcribed and translated.

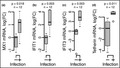

## 1 INTRODUCTION

The coronavirus disease 2019 (COVID‐19) pandemic due to severe acute respiratory syndrome (SARS) coronavirus‐(CoV)‐2 infection has afflicted millions following first reports (Timeline: WHO’s COVID‐19 response, 2020). Rapid molecular characterization of the virus (Corman et al., 2020; Lu, Zhao, et al., 2020; Wu et al., 2020) enabled multiple instances of successful containment using public health measures (Baker et al., 2020; Cheng et al., 2020; Cowling et al., 2020; Fouda et al., 2020; Kang et al., 2020; Wang et al., 2020). Elsewhere, however, viral spread has been rapid, widespread and devastating.

Silent infection with rapid viral replication allows asymptomatic person‐to‐person infection (Cheng et al., 2020; Kang et al., 2020; Sakurai et al., 2020; Wang, Ng, et al., 2020). Nasopharyngeal viral loads peak when upper airway symptoms appear (Cheng et al., 2020; Wölfel et al., 2020), and the size of the initial viral innoculum may determine the rapidity of onset and severity of the subsequent clinical syndrome (Gandhi et al., 2020). Over days or weeks, the infection may extend to involve the lower respiratory tract. For many patients, initial fever, dry cough, myalgias, and anosmia progress to dyspnea, hypoxemic respiratory failure, and the acute respiratory distress syndrome (ARDS) (Huang et al., 2020; Lescure et al., 2020; Wang, Hu, et al., 2020; Zhou, Yang, et al., 2020). Innate immune dysfunction and systemic inflammatory responses include marked elevations in systemic inflammatory cytokines and insufficient antiviral interferon (IFN) responses that create a cytokine storm (Chen et al., 2020; Pedersen & Ho, 2020; Wang et al., 2014; Ye et al., 2020; Zhou, Ren, et al., 2020).

Part of the challenge to developing a treatment or prevention response is to narrow the extensive list of potential biochemical treatment targets (Pedersen & Ho, 2020; Zhang et al., 2020). Evaluations in ARDS due to other severe respiratory viral infections (Chen & Subbarao, 2007; Gralinski & Baric, 2015; Jong et al., 2006; Kim et al., 2016; Kindler et al., 2013; Li & Lin, 2013; Nicholls et al., 2003; Qian et al., 2013; Wang et al., 2014) highlight the broad collection of biomarkers that may potentially be useful in SARS‐CoV‐2 infection.

Coronaviruses that cause severe human disease are remarkable for their ability to evade innate immune defenses and to promote dysfunctional responses that appear before cytokine storm (Lei & Hilgenfeld, 2017; Nelemans & Kikkert, 2019). For example, IFN responses are critically important for antiviral defense (Lazear et al., 2019), yet there is no detectable native human IFN response to SARS‐CoV (Zielecki et al., 2013). Both SARS‐CoV and SARS‐CoV‐2 stimulate inflammatory signals via nuclear factor κB (NFκB) (DeDiego et al., 2014) that recruit polymorphonuclear neutrophils and other immune effector cells to the lung, releasing proteases that may dramatically further increase viral cell entry (Heurich et al., 2014; Hoffmann et al., 2020; Matsuyama et al., 2005). IFN‐α and IFN‐β treatments that bypass some evasion strategies (Zhou, Ren, et al., 2020) have been proposed to counter SARS‐CoV‐2 infection (Mantlo et al., 2020). However, we lack efficacy and safety trials free of observer bias (Cox, 1958a), and no published human data exist for IFN‐λ therapy.

To supplement the growing information on responses early in infection, we undertook an observational study of deidentified nasopharyngeal swab samples from patients presenting at drive‐through testing centers for evaluation of symptoms potentially due to SARS‐CoV‐2 infection. We selected proteins involved in different steps of human cellular responses to viral invasion for quantitative measurements by multiple methods to understand the impact of targeting by viral evasion activities (DeDiego et al., 2014; Gralinski & Baric, 2015; Nelemans & Kikkert, 2019). We selected and measured factors important for understanding viral entry, intracellular detection of viral invasion, production of proinflammatory signals, systemic inflammatory agents, and multiple IFN and IFN‐stimulated gene (ISG) responses relative to viral loads to better understand the immune landscape of patients with early disease.

## 2 METHODS

### 2.1 Samples and study population

Our project was reviewed at the University of Utah by both the Institutional Review Board and the Biosafety Committee. An exemption from informed consent was allowed because patient samples were de‐identified. All samples were handled in a biosafety level (BSL) 2 capable hood (ThermoFisher Scientific, Waltham, MA, USA) using BSL 3 procedures until virus inactivation and were handled with BSL 2 procedures thereafter.

Randomly selected and completely deidentified, residual nasopharyngeal swab samples from patients presenting for diagnosis of symptoms consistent with COVID‐19 during the period of late April through early June of 2020 were included in the study. Clinical testing involved the use of a portion of each sample to test with automated, FDA Emergency Use Authorized real‐time polymerase chain reaction (RT‐PCR) or transcription‐mediated amplification tests for qualitative presence of SARS‐CoV‐2 RNA. We received sample remainders annotated with age, sex, and qualitative nucleic acid amplification‐detection results after being frozen at −80°C for approximately one month.

### 2.2 Initial extraction of human RNA and proteins

We extracted RNA using Chemagic reagents and Chemagic MSM I extraction platform (Perkin‐Elmer, Billerica, MA, USA) from part of each sample remainder producing sufficient RNA to allow RT‐PCR measurement of reference gene *Pol2A* (mean *CT =32*.22, SD =4.86). To exclude potential artifacts due to modified expression of *Pol2A* with viral infection, we measured *ActB* and *GAPDH* as alternative reference points and found no effects due to infection for any of the genes. *Pol2A* had the lowest standard error of measurements. Thus, for all other mRNA measurements, we used the Pol2A *C_T_* as the reference point to calculate fold change (see Methods for *C_T_* definition and usage).

Protease inhibitor cocktail and equal volume of Hank's Balanced Salt solution (Sigma Aldrich) were added to the final portion of the thawed patient samples prior to centrifugation (20,000 g for 20 min at 4°C). We carefully aspirated the supernatant for Bead Based Multiplex Immunoassays. Pellets from centrifugation were extracted using All‐Prep Micro kits (Qiagen) in accordance with the manufacturer's instructions, producing additional RNA suitable for RT‐PCR and a final protein‐containing pellet for Mass Spectrometry.

### 2.3 Real‐time polymerase chain reaction of viral and human mRNA

For most mRNAs, we had a sufficient samples to study all 40 patients; for selected mRNAs, we were able to study six samples with and six samples without SARS‐CoV‐2 detection. All specific mRNA measurements were based on RT‐PCR employing RNA from a single extraction method to avoid technical sources of noise.

An equal amount of RNA was taken for first‐strand cDNA reverse transcription (ABI High Capacity cDNA Reverse Transcription Kit) and specific amplification in a StepOnePlus (ABI, ThermoFisher Scientific). Gene‐specific primers were designed using the Roche Applied Science Universal Probe Library Assay Design Center. All amplifications were performed using a 2‐step amplification protocol with ABI PowerUp SYBR Green Master Mix as follows: 1 cycle at 50°C for 2 minutes to activate UDG, 1 cycle at 95°C for 2 minutes to release the DNA polymerase then 40–50 cycles with a 3‐second denaturing at 95°C followed by 30‐second annealing and denaturing at 60°C.

A melt curve (dissociation) was performed for every primer to ensure the above amplification conditions resulted in the amplification of a single peak. All of the designed primers gave a single peak upon dissociation after amplification suggesting no nonspecific binding to other genes. Amplification of genomic DNA was prevented by using primers that spanned an intron. The *IFN*‐*α2* gene and *IFITM*‐*1* ISG do not have introns. The primers did, however, give a single peak upon dissociation. All other primers including *IFN*‐*λ* spanned an intron.

### 2.4 Bead based multiplex immunoassays

Cytokine analyses of patient samples were performed using a commercially available enzyme‐linked immunosorbent assay (LEGENDplex Human Anti‐Virus Response Panel 13‐Plex with Filter Plate, BioLegend, San Diego, CA). This bead‐based multiplex assay allowed for the simultaneous quantification of interleukins (IL‐1β, IL‐6, IL‐8 [or CXCL8], IL‐10, IL‐12p70); interferons (IFN‐α2, IFN‐β, IFN‐γ, IFN‐λ1, IFN‐λ2,3), TNF‐α, IP‐10 (or CXCL10), and GM‐CSF in patient samples using a flow cytometric approach. All standards and samples were assayed in duplicate using manufacturer recommended protocols. Incubation steps were conducted at room temperature with constant agitation (500 rpm), and shielded from exposure to light. Performing the assay in standard 96‐well filter plates facilitated thorough washing of samples and required the use of a MultiScreen Vacuum Manifold (EMD Millipore Corporation, Billerica, MA) alongside a uniform vacuum source. Following the final wash of combined sample and biotinylated detection antibodies, bound proteins of interest were re‐suspended in 0.008% final concentration of EM‐grade glutaraldehyde (Electron Microscopy Sciences, Hatfield, PA, USA, Cat #16216) for 48 hours at 4°C to inactivate SARS‐CoV‐2, adapting a protocol previously investigated for SARS‐CoV (Darnell et al., 2004). Following incubation, samples were washed a final time and transferred to a polystyrene 96‐well plate with conical bottoms. Flow cytometric analysis of cytokines was performed using a BD FACSCanto II system (BD Biosciences, San Jose, CA) at the University of Utah Flow Cytometry Core (Salt Lake City, UT) and analyzed using LEGENDplex software (BioLegend).

### 2.5 Mass spectrometry

#### 2.5.1 Preparation of proteins prior to mass spectrometry

Proteins were reduced with 5 mM dithiothreitol (DTT) at 60°C for 45 minutes, followed by alkylation with 10 mM iodoacetamide (IAA) at room temperature for 30 minutes in the dark. Excess IAA was neutralized by the addition of 5 mM DTT. A trypsin/LysC mixture (Promega; Madison, WI) was added to the proteins in a 1:100 ratio and the proteins were digested overnight at 38°C. The digestion was quenched by acidification of the solution with the addition of 1% formic acid to a pH of 2–3.

Initially, the pelleted proteins from the COVID‐19 patients would not completely dissolve in 50 mM ammonium bicarbonate. However, after the trypsin/LysC digestion, all of the samples were completely dissolved in solution. The final concentration of the peptides was determined using a peptide colorimetric assay and the use of a Nanodrop One (ThermoFisher Scientific) spectrophotometer.

#### 2.5.2 Data‐Dependent Acquisition (DDA) nanoLC‐MS/MS

Peptides (1 μg on column) were loaded using a Dionex UltiMate 3000 RSLCnano system (ThermoFisher Scientific) onto a PharmaFluidics μPAC micro‐chip based trapping column and separated using a 50 cm equivalent PharmaFluidics μPAC microchip‐based column (PharmaFluidics, Ghent, Belgium). Chromatography was performed using ultrapure water with 0.1% formic acid (solvent A) and acetonitrile containing 0.1% formic acid (solvent B). Elution was carried out with an initial mobile phase concentration of 5% for 4 minutes followed by a ramp to 45% over 76 minutes then a second ramp to 95% B in 5 minutes. This was held for 10 minutes followed by ramping down to 5% B over two minutes and re‐equilibration for 10 minutes. Flowrate was 0.5 mL/min. A QExactive HF (ThermoFisher Scientific) coupled to a Flex nanospray source was employed with the following settings for MS1; resolution 60, AGC target 3e6, maximum IT 100 ms, scan range 375–1650 m/z. MS2 settings were as follows: resolution 15,000, AGC target 2e5, maximum IT 25 ms, isolation window 1.4 m/z. Top 15 DDA analysis was performed with NCE set to 27.

#### 2.5.3 Data‐Independent Acquisition (DIA) nanoLC‐MS/MS

Staggered window DIA analysis was carried out using the methods described by (Pino et al., 2020). A peptide centric gas phase retention time library was generated by pooling equal amounts of each sample and analyzing this using six narrow windows. DIA experiments with the following settings for MS1: resolution 60,000, AGC target 1e6, maximum IT 55, with six separate analyses in the following mass ranges 395–505 m/z, 495–605 m/z, 595–705 m/z, 695–805 m/z, 795–905 m/z, and 895–1005 m/z. MS2 analysis used the following settings: resolution 30,000, AGC target 1e6, loop count 25, default charge 3, NCE 27 with 4 m/z staggered DIA windows. NanoLC‐MS/MS analysis was carried out identically to DDA analysis described above.

#### 2.5.4 DDA data processing

The Proteome Discover version 2.4 (ThermoFisher Scientific) precursor‐based quantification processing workflow was employed. SequestHT with multiple peptide search and percolator validation was employed to extract protein data. The following search options were employed, *Homo sapiens* FASTA file, trypsin digestion, two missed cleavages, minimum peptide length 6, precursor mass tolerance 10 ppm, fragment mass tolerance 0.02 Da, carbamidomethylation of cysteine as a peptide static modification, N‐terminal acetylation, N‐terminal Met‐loss and methionine oxidation as protein dynamic modifications.

#### 2.5.5 DIA data processing

Thermo .RAW files were demultiplexed and converted to mzML files using MSConvert (Chambers et al., 2012). The Walnut functionality of EnclopeDIA (Searle et al., 2018) was employed for peptide centric library creation. Peptides were identified using the same variables as DDA described above. Quantitation was performed using Skyline (MacLean et al., 2010).

### 2.6 Calculations and statistical analysis

For all mRNA, we calculated fold‐change for each sample (*FCsample*) after measuring the fractional number of polymerase chain reaction doubling cycles required so that SYBR Green fluorescence exceeded the threshold for detection (*C_T_*). We used the following formula:

$$FCsample=2^{‐\left(\Delta C_{T}sample‐\Delta C_{T}median\right)}$$

where *ΔC_T_sample* was the number of doubling cycles to detect each mRNA minus the number of doubling cycles to detect mRNA from the *Pol2A* reference gene for each sample, and the *ΔC_T_median* was the median *ΔC_T_sample* for samples without detection of SARS‐CoV‐2. The incorporation of *C_T_* for *Pol2A* mRNA in the calculation indexes the measurement so that samples with different efficiencies of recovery of mRNA containing cells between testing individuals are standardized.

For IFIT‐3 fold change values from DIA mass spectrometry, *FCsample* was calculated:

$$FCsample=\frac{AUC_{IFIT3}}{median\left(AUC_{virusnegativeIFIT3}\right)},$$

where *AUC_IFIT3_* is the area under the curve (AUC) for peptides identified as part of IFIT‐3, and *median*(*AUC_virus negative IFIT3_*) is the median AUC for IFIT‐3 from samples without detection of SARS‐CoV‐2. The other prespecified ISG proteins were undetectable by DIA mass spectrometry, and thus no fold‐change calculation was possible.

We calculated summary statistics. We examined associations between different biomarker measurements by calculating Spearman's rank correlation statistic to better understand potential dependencies. We used log transformations of all mRNA and protein measurements in our statistical calculations because of the log‐normal nature of our results. Others using the methods that we employed, however, often report results using either natural or base 2 logs. Because the bulk of our results are commonly reported using natural log values, we standardized on those for reporting. The effect is to slightly change results normally reported using base 2 logs by a proportion equal to natural log of 2 (or 0.698). This usage has no effect on interpretations of results.

Using SARS‐CoV‐2 infection status as the independent variable, we performed linear regression with natural log(*FC*) of each prespecified mRNA or natural log(*FC_IFIT3_*) as the dependent variable because we seek to understand the biological effects of infection. Each univariable model was adjusted with age and sex with backward selection to understand the impacts on model fits. We performed univariable linear regression with natural log(*FC*) or natural log(protein concentration [pg/ml]) as the dependent variables and natural log(*FC_viral N1 protein mRNA_*) as the independent variable to understand associations with viral load, adjusting with age and sex as above.

We performed sensitivity analyses of significant associations. For each dependent biomarker with significant associations with infection status or viral load, we selected all other biomarkers reported to have significant correlations as additional adjustment variables. Using these adjustment biomarkers one at a time, we assessed the impact on the estimates for infection status and viral load for each significant association.

We assigned 50 as the *C_T_* value for undetectable mRNA. For undetectable proteins by bead‐based multiplex immunoassay, we assigned the minimum detection value. These assignments enable quantitative analysis without treating the values as missing. Results were similar when the analysis was restricted to raw data derived from the six infected and six uninfected samples with the highest recovery of RNA. All calculations and statistical modeling were performed using the R statistical system (R Core Team, 2020).

## 3 RESULTS

### 3.1 Study population

**FIGURE 1:**
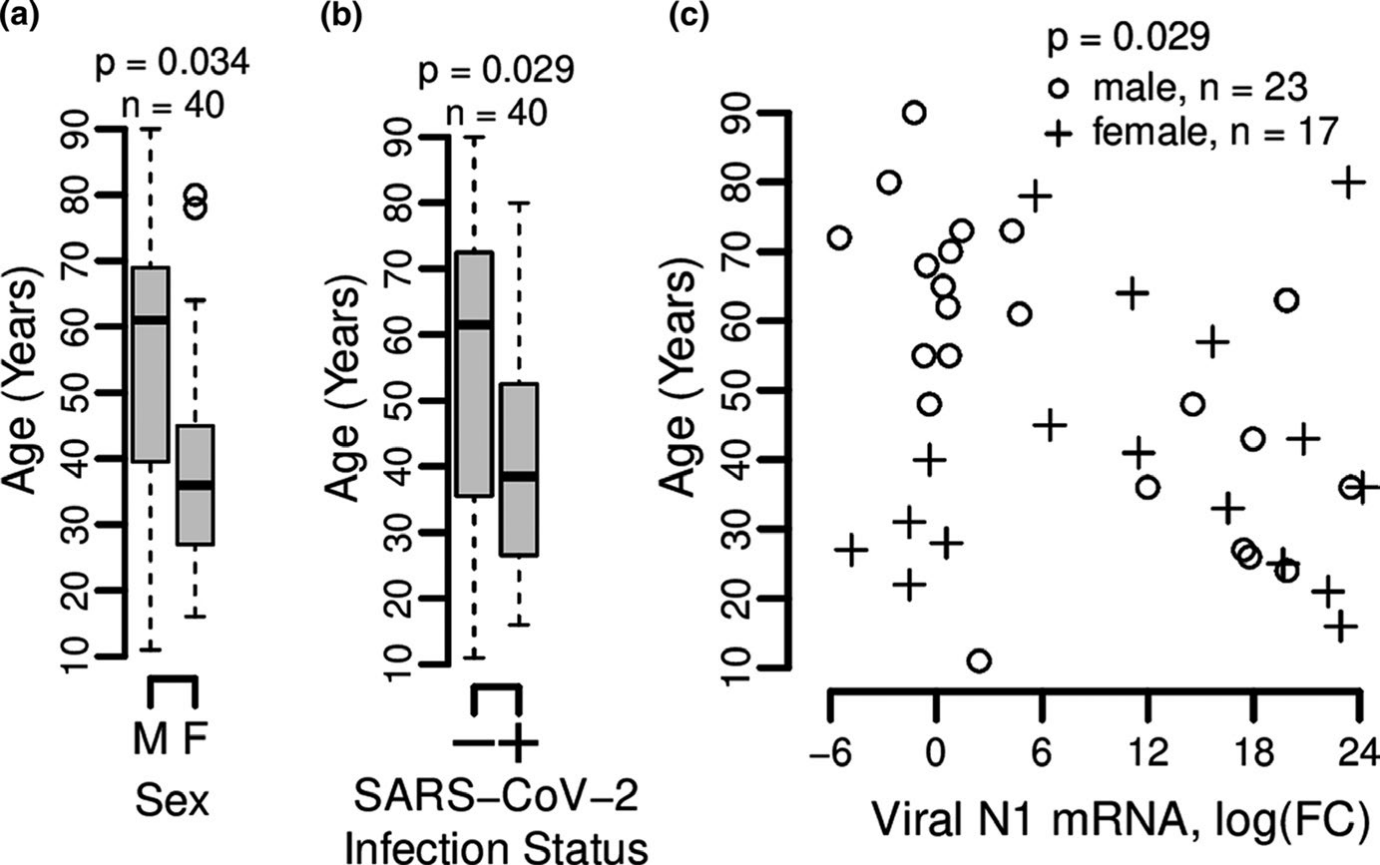
Relationships with Age. In *A*: male patients were older and in *B*: patients without infection were older, but in *C*: this relationship is seen to be limited to male patients (see also Table 3). Each box‐plot includes boxes that show median, upper, and lower quartile values and whiskers or single points that show upper and lower extremes (McGill et al., 1978);*p* values were calculated using linear regression (Chambers, 1998).

We evaluated 40 samples from individuals, evenly divided into 20 positive and 20 negative detection results for SARS‐CoV‐2. Samples were deidentified but annotated by age (median 46.5 years, range 11–90) and sex (17 females, 42.5%). Older patients were more likely to be male and negative for SARS‐CoV‐2 detection (Figure 1 and Tables 1, 2, 3). This limited demographic information suggested that further evaluation of statistical relationships in our sample set required testing adjustments for age, sex, or both to avoid confounding.

**TABLE 1:** Demographics.

Sex	Age Range and SARS‐CoV−2 Status		n
	Not Detected	Detected	
male	11–90	24–63	23
female	22–78	16–80	17
n	20	20	40

**TABLE 2:** Relationships Between Sex and Virus Detection Status with Age^a^.

Age Effect	Estimate	SE	*t*	OR	95% CI	*p*
Female	−0.0361	0.0177	−2.04	0.965	0.932–0.999	0.041
SARS2 Detected	−0.0365	0.0174	−2.09	0.964	0.932–0.998	0.036

^a^ Univariable logistic regression models (Cox, 1958b; Hosmer et al., 2013) of age as the input variable with female sex and positive virus detection as output variables. Younger patients are more likely to be female and more likely to be infected.

**TABLE 3:** Relationship of Age with Viral Load by Sex^a^.

Sex	Estimate	SE	*t*	OR	95% CI	*p*
Male	−1.22	0.39	−3.14	0.294	0.137–0.631	0.005^†^
Female	0.195	0.482	0.404	1.21	0.472–3.12	0.692

^a^ Univariable linear regressions between Age in years as the dependent variable and log(FC) of viral N1 protein for each sex.

Samples included in our study were randomly selected from those collected from April‐June of 2020 from patients who may have come from nine states within the Mountain West of the United States. During this period, positive results were reported for about 9–10% of tested patients. Among the positive test patients, about 10% eventually required hospitalization for COVID‐19 with less than 50% of those hospitalized suffering respiratory failure, ARDS, or succumbing to severe disease. While we know this context for our samples, the specific outcomes for individual patients in our study remain unknown.

### 3.2 Viral load

Using RT‐PCR, we estimated fold‐change in mRNA expression of SARS‐CoV‐2 small envelope protein E1 (Odds Ratio [OR] =10.8 × 10^6^, 95% Confidence Interval [CI] =8.37 × 10^5^–1.40 × 10^8^, *p* < 0.001) and nucleocapsid protein N1 (OR =5.1 × 10^7^, CI =4.5 × 10^6^–5.9 × 10^8^, *p* < 0.001) relative to expression in patients without infection. We selected primers (Udugama et al., 2020) for *E1* originally from Charité, Germany (Corman et al., 2020) and *N1* from the US CDC (Lu, Wang, et al., 2020). Both *E1* and *N1* mRNA fold changes gave virtually total discrimination between patients with and without infection diagnosed by clinical testing for SARS‐CoV‐2 infection using qualitative RT‐PCR.

### 3.3 Viral entry

**FIGURE 2:**
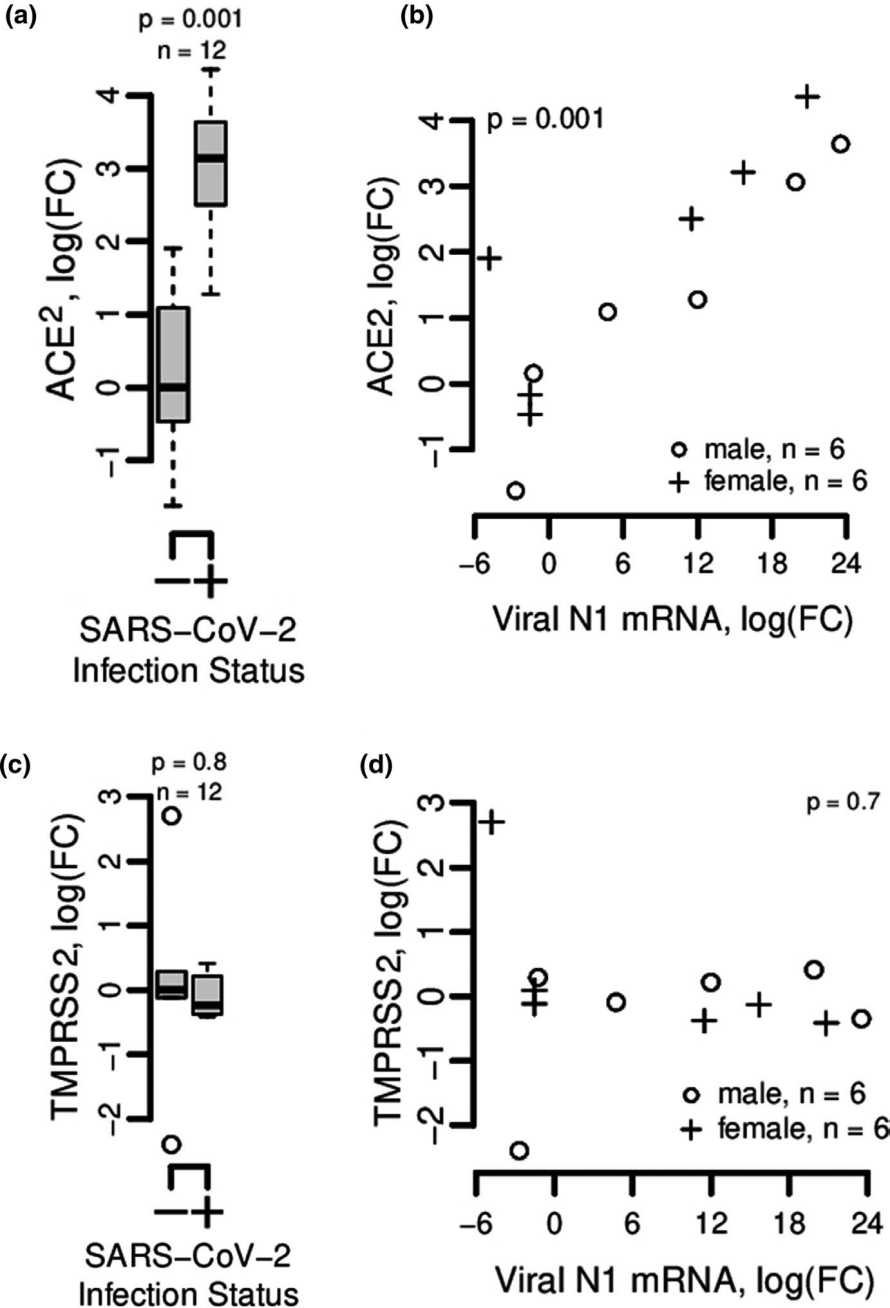
Association of *ACE2* but not *TMPRSS2* Expression with SARS‐CoV‐2 Infection. *A*: *ACE*‐*2* mRNA is increased approximately three‐fold in patients with SARS‐CoV‐2 infection over *ACE*‐*2* mRNA expression in patients without infection, and *B*: the increase in expression is associated with viral load (OR =1.16, CI =1.1–1.23, *p* < 0.001). However, the expression of *TMPRSS*‐*2* is *C*: neither increased nor decreased with infection and *D*: is not associated with viral load. Adjustments for age and sex were not significant for either molecule. In each panel, *A*‐*D*, there are six infected and six noninfected status patients. Each box‐plot includes boxes that show median, upper, and lower quartile values and whiskers or single points that show upper and lower extremes (McGill et al., 1978), and *p* values were calculated using linear regression (Chambers, 1998).

We measured two human protein transcripts important for understanding SARS‐CoV‐2 cell entry, *angiotensin*‐*converting enzyme*‐*2* (*ACE*‐*2*), which is essential for entry of SARS‐CoV‐2 and SARS‐CoV (Hoffmann et al., 2020; Shang et al., 2020), and *transmembrane protease*, *serine*‐*2* (*TMPRSS*‐*2*) which enhances cell entry up to a thousand‐fold (Heurich et al., 2014; Hoffmann et al., 2020). *ACE*‐*2* mRNA was increased threefold in patients with infection, and the fold‐change results were strongly associated with viral load. *TMPRSS*‐*2* mRNA expression, however, was not associated with infection nor viral load (Figure 2).

### 3.4 Viral detection signaling

We examined transcription signals for two genes in the signaling pathway downstream of viral detection important for IFN responses, *TNF*‐*associated factor*‐*binding kinase*‐*1 associated with inhibitor of NFκB* (*TBK*‐*1*) and *Stimulator of IFN genes*‐(*STING*)‐*1* for six patients with positive detection of SARS‐CoV‐2 and six patients with negative detection. The mRNA expressions of *TBK*‐*1* and *STING*‐*1* were not associated with infection.

### 3.5 Inflammatory responses to SARS‐CoV‐2 infection

**FIGURE 3:**
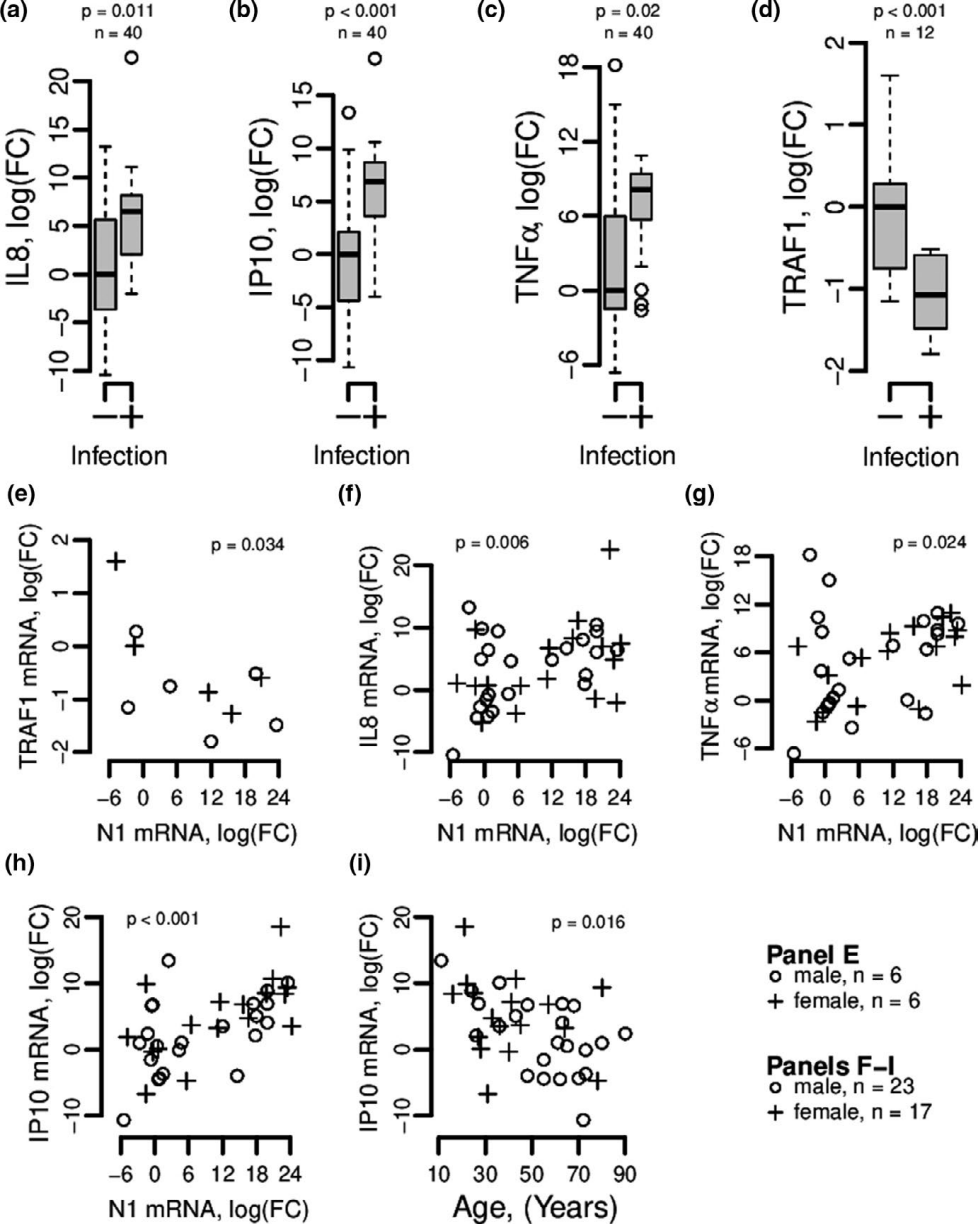
Transcripts for Genes Associated with Inflammation. Transcription of *A*: *IL*‐*8*, *B*: *IP*‐*10*, *C*: *TNF*‐*α* mRNA are all increased approximately sixfold, whereas transcription of *D*: *TRAF*‐*1* is reduced. When assessed for relationship with viral load, *E*: decreasing *TRAF*‐*1* mRNA is associated with increasing viral *N1* protein mRNA, whereas increasing mRNA for *F*: *IL*‐*8*, *G*: *TNF*‐*α* (less strongly) and *H*: *IP*‐*10* are associated with increasing viral *N1* protein mRNA. In contrast, *I*: decreasing *IP*‐*10* mRNA is associated with increasing age. Similar relationships are seen in viral *E1* protein transcripts (not shown). In each panel, *A*‐*C*, there are 20 infected and 20 noninfected status patients. In panel *D*, there are six infected and six noninfected. Each box‐plot includes boxes that show median, upper, and lower quartile values and whiskers or single points that show upper and lower extremes (McGill et al., 1978), and *p* values were calculated using linear regression (Chambers, 1998).

We found increased mRNA expression of *IL*‐*8*, *IFN*‐*γ*‐*induced protein*‐(*IP*)‐*10*, and *TNF*‐*α* in SARS‐CoV‐2‐infected individuals (Figure 3A‐C). Moreover, we found that there was a strong association between viral load and the level of mRNA expression of these innate immune effector molecules (Figure 3F‐H).

Viral entry, detection, and signaling may lead to a systemic inflammatory response via *NFκB* activity which may potentially be augmented by *TNF receptor*‐*associated factor*‐(*TRAF*)‐*1* activity (Edilova et al., 2018; Lalani et al., 2018). We found a large reduction in *TRAF*‐*1* mRNA (Figure 3D). The reduction in *TRAF*‐*1* was inversely associated with expression of both viral protein *E1* mRNA (OR =0.945, CI =0.906–0.986, *p* = 0.025) and *N1* mRNA (OR =0.947, CI =0.907–0.989, *p* = 0.034, Figure 3E). We found *NFκB*‐*1* and *NFκB*‐*2* mRNA transcripts were not significantly changed compared to uninfected status (Table 4). Other downstream immune effectors, *granulocyte*‐*macrophage colony*‐*stimulating factor* (*GM*‐*CSF*), *IL*‐*6* and *IL*‐*10* mRNA were not increased (Table 4).

**TABLE 4:** Systemic Inflammatory mRNA Response to SARS‐CoV‐2 Infection.

mRNA^a^	Estimate	SE	t	OR	CI	*p*
*GM‐CSF*	0.136	1.74	0.0784	1.15	0.038–34.6	0.94
*IL−6*	−1.21	2.36	−0.514	0.298	0.0029–30.1	0.61
*IL−8*	4.93	1.83	2.69	139	3.82–5030	0.011
*IL−10*	3.94	2.01	1.97	51.5	1.01–2620	0.057
*IP*−*10* ^b^	4.87	1.62	3.00	131	5.43–3160	0.005
*NFκB−1*	1.4	0.956	1.47	4.07	0.625–26.5	0.17
*NFκB−2*	1.58	2.03	0.778	4.84	0.0909–258	0.46
*TNF‐α*	4.09	1.67	2.44	59.7	2.24–1590	0.019
*TRAF1*	−1.08	0.442	−2.45	0.339	0.143–0.805	0.034

^a^ Results in alphabetical order of mRNA names show natural log(fold‐change in mRNA expression) as the dependent variable and clinical viral detection as the independent variable. Adjustments for age and sex were not significant except as noted. All results were calculated using linear regression (Chambers, 1998).

^b^ Adjustment for sex was not significant, but patients had decreased IP‐10 mRNA for each year of additional age (OR =0.91, 95% CI =0.84–0.98, *p* = 0.016).

Protein measurements using bead‐based multiplex immunoassays (BioLegend) for systemic inflammatory markers matching many of the mRNAs measured (plus IL‐1b and IL‐12p70) revealed no significant changes with infection and no significant associations with viral load with one exception. IP‐10 protein was increased nearly fourfold above measured control values (Table 5), and the log of concentration was strongly associated with viral load (OR =1.09 per unit of log unit of viral *N1* mRNA fold‐change, CI =1.06–1.12, *p* < 0.001).

**TABLE 5:** Protein production of inflammatory markers (log[pg/ml]) with SARS‐CoV‐2 Infection.

Protein^a^	Estimate	SE	t	OR	CI	*p*
GM‐CSF	−0.00644	0.0060	−1.07	0.994	0.982–1.01	0.29
IL−1b	−0.0594	0.0358	−1.66	0.942	0.878–1.01	0.10
IL−6	0.116	0.113	1.02	1.12	0.899–1.4	0.32
IL−8	0.0976	0.457	0.214	1.1	0.45–2.7	0.83
IL−10	0.106	0.0846	1.25	1.11	0.942–1.31	0.22
IL−12p70	−0.0888	0.078	−1.14	0.915	0.785–1.07	0.26
IP−10	1.32	0.302	4.36	3.74	2.07–6.77	<0.001
TNF‐α	0.0265	0.0265	1.00	1.03	0.975–1.08	0.32

^a^ Results show natural log(pg/ml) of each protein as a function of viral detection. Adjustments for age and sex were not significant. All results were calculated using linear regression (Chambers, 1998).

### 3.6 IFN responses to infection

**FIGURE 4:**
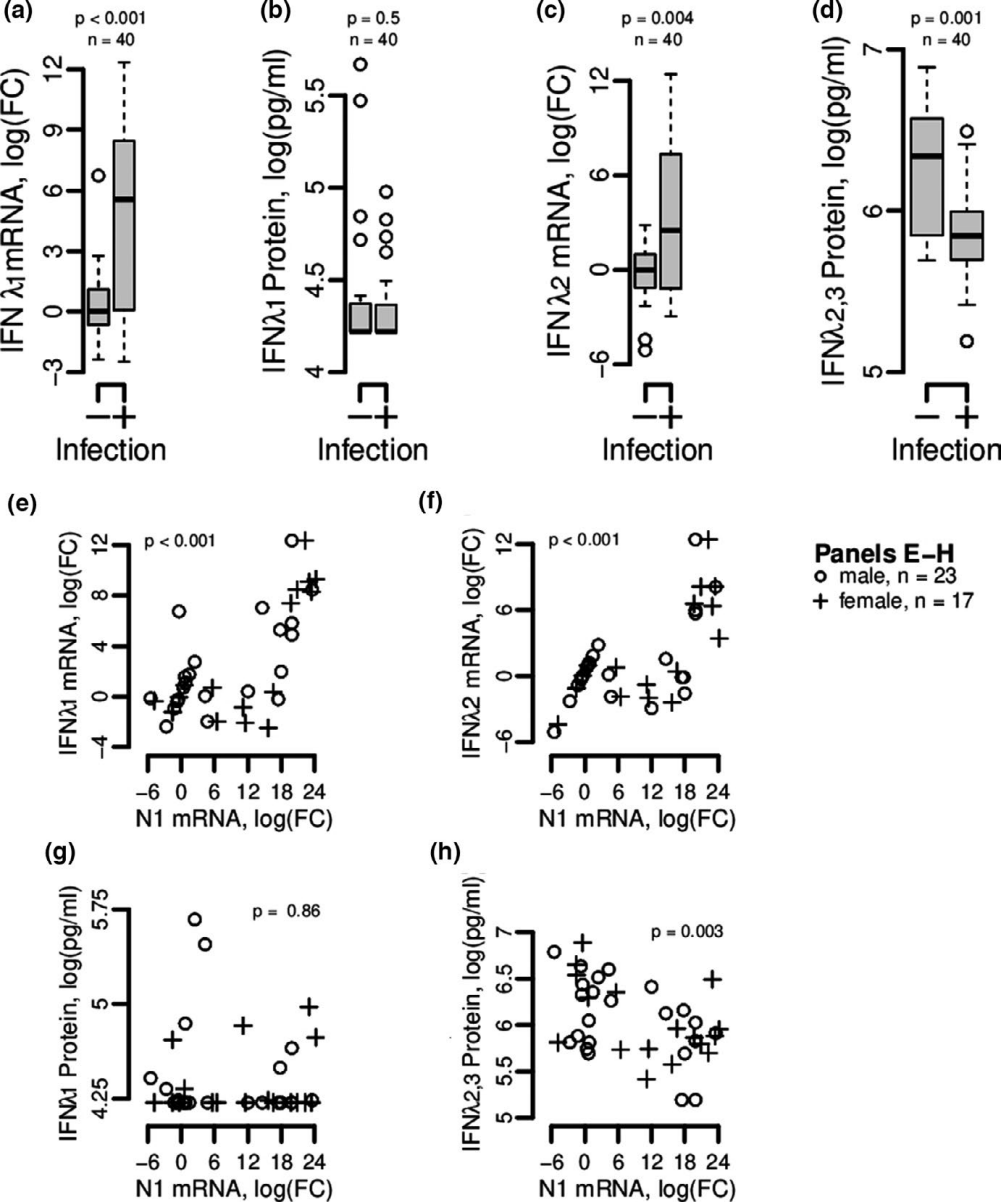
Transcripts for Genes Associated with Interferons. Transcription of *A*: *IFN*‐*λ1* is increased nearly sixfold although *B*: IFN‐λ1 protein is unchanged with infection. Transcription of *C*: *IFN*‐*λ2* is increased about 3‐fold but *D*: IFN‐λ2 protein is decreased even if the combined measurement of IFN‐λ2 and IFN‐λ3 proteins are attributed solely to IFN‐λ2. The mRNA fold changes for *E*: *IFN*‐*λ1* and *F*: *IFN*‐*λ2* are directly associated with increasing viral *N1* protein mRNA. For *G*: IFN‐λ1 protein there is no association with viral *N1* protein mRNA, but for *H*: combined IFN‐λ2 and IFN‐λ3 protein measurement, there is an inverse relationship with viral *N1* protein mRNA. For *E*‐*H*, substitution of viral *E1* mRNA produced similar relationships and figures (not shown). In each panel, *A*‐*D*, there are 20 infected and 20 noninfected status patients. Each box‐plot includes boxes that show median, upper, and lower quartile values and whiskers or single points that show upper and lower extremes (McGill et al., 1978), and *p* values were calculated using linear regression (Chambers, 1998).

We found five to sixfold increases in expression of both *IFN*‐*λ1* and *IFN*‐*λ2* mRNA among patients with detection of SARS‐CoV‐2 (n = 20) compared to patients without detection of virus (n = 20) (Table 6, Figure 4A,C). There were no other significant increases in *IFN* mRNA. The increases in *IFN*‐*λ1* and *IFN*‐*λ2* mRNA production were strongly associated with viral load (Table 7 and Figure 4E,F).

**TABLE 6:** IFN Response to SARS‐CoV‐2 Infection.

mRNA^a^	Estimate^†^	SE	*t*	OR	CI	*p*
*IFN‐α2*	1.17	0.993	1.17	3.21	0.458–22.5	0.25
*IFN‐β1*	0.88	1.16	0.756	2.41	0.246–23.6	0.45
*IFN‐γ*	1.89	1.03	1.84	6.65	0.883–50.1	0.074
*IFN‐λ1*	4.26	1.18	3.62	71	7.07–713	<0.001
*IFN‐λ2*	3.69	1.2	3.09	40.2	3.86–419	0.004
*IFN‐λ3*	−0.991	2.67	−0.371	0.371	0.00196–70.2	0.71

^a^ Results are show natural log(fold‐change in mRNA expression) as a function of viral detection. Adjustments for age and sex were not significant for any model. All results were calculated using linear regression (Chambers, 1998).

**TABLE 7:** IFN response associations with viral load (N1 protein).

mRNA^a^	Estimate	SE	*t*	OR	CI	*p*
*IFN‐α2*	0.102	0.0495	2.06	1.11	1.01–1.22	0.046
*IFN‐β1*	0.0904	0.0588	1.54	1.09	0.975–1.23	0.13
*IFN‐γ*	0.14	0.0507	2.75	1.15	1.04–1.27	0.009
*IFN‐λ1*	0.302	0.0507	5.96	1.35	1.23–1.49	<0.001
*IFN‐λ2*	0.285	0.0515	5.53	1.33	1.2–1.47	<0.001
*IFN‐λ3*	0.0531	0.138	0.384	1.05	0.804–1.38	0.70

^a^ Results show linear regression with natural log(fold‐change in mRNA expression) as the dependent variables and natural log(viral *N1*) detection as the independent variable. Adjustments for sex and age were not significant for any IFN. All results were calculated using linear regression (Chambers, 1998).

Despite increased mRNA expression for some of the IFNs, protein measurements showed reductions in IFN‐α2, IFN‐γ, and IFN‐λ2,3 in patients with viral infection that averaged 66%, 49%, and 40%, respectively, relative to control patients (Table 8).

**TABLE 8:** IFN Protein Production with SARS‐CoV‐2 Infection.

Protein^a^	Estimate	SE	t	OR	CI	*p*
IFN‐α2	−1.08	0.428	−2.53	0.338	0.146–0.783	0.016
IFN‐β	−0.0957	0.0838	−1.14	0.909	0.771–1.07	0.26
IFN‐γ	−0.672	0.222	−3.02	0.511	0.33–0.79	0.004
IFN‐λ1	−0.0813	0.11	−0.74	0.922	0.743–1.14	0.46
IFN‐λ2,3^b^	−0.520	0.115	−4.52	0.595	0.475–0.745	<0.001

^a^ Results show log(pg/ml) of each protein as a function of viral detection. Adjustments for sex and age were not significant except as noted. All results were calculated using linear regression (Chambers, 1998).

^b^ Adjustment for sex was not significant, but patients had slightly decreased IFN‐λ2,3 in response to SARS‐CoV‐2 infection for each additional year of age (OR =0.99, 95% CI =0.988–0.999, *p* = 0.048).

### 3.7 ISG responses to infection

**FIGURE 5:**
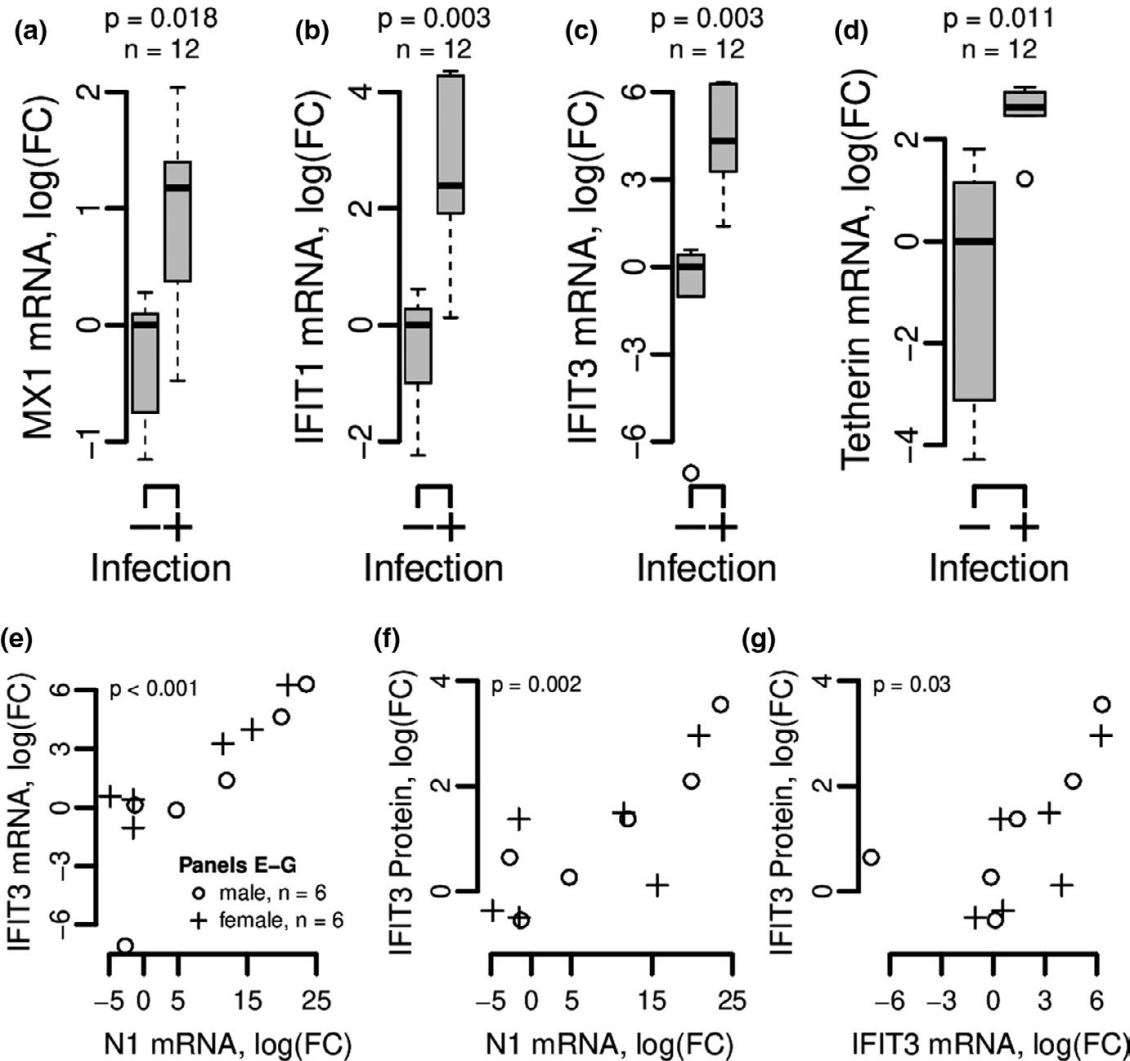
Transcripts for Pre‐selected ISGs. Infection with SARS‐CoV‐2 is associated with increased *A*: *MX*‐*1*, *B*: *IFIT*‐*1*, *C*: *IFIT*‐*3*, and *D*: *Tetherin* (*BST*‐*2*) mRNAs. There were significant associations between increasing *E*: *IFIT*‐*3* mRNA and *F*: IFIT‐3 protein fold changes and increasing viral *N1* protein mRNA. Protein fold‐change for IFIT‐3 was measured using DDA mass spectrometry. Similar relationships were seen using viral *E1* protein mRNA as in *E* and *F*. In each panel, *A*‐*F*, there are six infected and six noninfected status patients. Each box‐plot includes boxes that show median, upper, and lower quartile values and whiskers or single points that show upper and lower extremes (McGill et al., 1978), and *p* values were calculated using linear regression (Chambers, 1998).

We prospectively selected four ISGs to evaluate because of their importance in defense against RNA viruses (Sadler & Williams, 2008): GTP‐binding Myxovirus protein (*MX*‐*1*) (Haller et al., 2015; Hefti et al., 1999; Kochs & Haller, 1999; Kochs et al., 2002; Verhelst et al., 2013). IFN‐induced proteins with tetratricopeptide repeats (*IFIT*) (Diamond & Farzan, 2013), IFN‐induced transmembrane protein (*IFITM*) (Diamond & Farzan, 2013; Perreira et al., 2013), and Tetherin (*BST*‐*2*) (Blanco‐Melo et al., 2016; Wang et al., 2019). *MX*‐*1* and *Tetherin* mRNA were both increased in infected patients with moderate statistical significance, whereas two *IFIT* mRNAs were greatly and significantly increased (Table 9). Focused proteomic examination of proteins extracted from samples using DDA mass spectrometry detected an association between positive clinical testing for SARS‐CoV‐2 and IFIT‐1, IFIT‐3, and Tetherin proteins with a borderline finding for MX‐1 protein (Table 10). Proteomic examination using DIA mass spectrometry, which has less sensitivity but better specificity and precision, detected only a large increase in IFIT‐3 protein that was associated with clinical infection detection and increasing viral *N1* mRNA (Figure 5). Transcript and proteome results are based on the same six positive and six negative samples for which we had sufficient mRNA remaining after other studies.

**TABLE 9:** IFN‐stimulated gene transcript responses with SARS‐CoV‐2 Infection.

mRNA^a^	Estimate	SE	t	OR	CI	*p*
*BST*−*2* ^b^	3.22	1.03	3.13	25.1	3.33–188	0.011
*IFIT−1*	2.97	0.777	3.82	19.5	4.25–89.2	0.003
*IFIT−3*	5.5	1.43	3.86	245	15–4020	0.003
*IFITM−1*	0.601	0.921	0.653	1.82	0.3–11.1	0.53
*MX−1*	1.2	0.427	2.82	3.33	1.44–7.7	0.018

^a^ Results show log(fold change of each mRNA) as a function of viral detection. Adjustments for age and sex were not significant. All results were calculated using linear regression (Chambers, 1998).

^b^ *BST*‐*2* is also known as *Tetherin*.

**TABLE 10:** IFN‐Stimulated Proteins Using Data‐Dependent Acquisition by Mass Spectrometry.

Protein^a^	χ^2^	*p*
BST−2	5.48	0.019
IFIT−1	8.33	0.004
IFIT−3	8.33	0.004
IFITM−1^b^	–	–
MX−1	3.375	0.066

^a^ χ^2^ tests (Karl Pearson, 1900) were applied to 2 × 2 tables of detection of protein vs detection of SARS‐CoV‐2 in all cases except for IFIT‐1. There was the detection of IFIT‐1 in nearly all samples, however, they segregated into high level or low‐level detection, and the χ^2^ test was applied to a 2 × 2 table of high detection of protein vs detection of SARS‐CoV‐2. For each result shown, there were n = 6 SARS‐CoV‐2 negative and n = 6 SARS‐CoV‐2 positive patients.

^b^ Not Detected.

### 3.8 Model adjustments with older age

The increase in *IP*‐*10* mRNA with infection was lower in older individuals (Figure 3I). All other things being equal, 10 additional years in age were associated with an approximately overall 70% reduction in *IP*‐*10* mRNA compared to controls, whereas 25 additional years in age were associated with an overall 90% reduction, producing a remarkable counter effect to the infection itself.

We found a borderline significant effect for IFN‐λ2,3. Patients with older ages have slightly lower IFN‐λ2,3 per additional year of age with SARS‐CoV‐2 infection. This small per year effect was associated with a 10% lower IFN‐λ2,3 on average for every additional 10 years of age and about 23% lower IFN‐λ2,3 for an additional 25 years of age in addition to the approximately 40% reduction associated with SARS‐CoV‐2 infection (Table 8).

### 3.9 Correlations between IFN, inflammatory and ISG measurements

We found no strong and significant correlations between any protein and its transcript suggesting widespread translation blockade or rapid protein degradation with two exceptions. Both *IP*‐*10* mRNA and protein were directly associated with viral load (*N1* protein mRNA), and the correlation between mRNA and protein was moderate with strong significance, *p *<* *0.001 (Table 12). *IFIT*‐*3* mRNA and protein were strongly associated with viral load (Figure 5E‐F) but somewhat weakly correlated with each other (Figure 5G and Table 12). In contrast, we found strong correlations within IFN and inflammatory proteins (Table 11) and within IFN gene, inflammatory signaling gene and ISG transcripts (Table 13). Correlations within IFN types, for example, among IFN‐λ subtypes were exceptionally strong which corresponds to the biology of the IFNs.

**TABLE 11:** Correlations among measured protein biomarkers^a^.

Protein, log(pg/ml)^b^	Protein, log(pg/ml)												
	GMCSF	IFNα2	IFNβ	IFNγ	IFNλ1	IFNλ23	IL1β	IL6	IL8	IL10	IL12p70	IP10	TNFα
IFNα2	‐												
**IFNβ**	0.33	**0.7**											
**IFNγ**	0.35	**0.72**	**0.62**										
**IFNλ1**	‐	‐	‐	‐									
**IFNλ23**	0.33	**0.53**	0.46	**0.63**	‐								
**IL1β**	‐	**0.61**	0.35	0.42	‐	0.36							
**IL6**	‐	‐	0.32	‐	‐	‐	‐						
**IL8**	‐	‐	‐	‐	‐	‐	‐	0.4					
**IL10**	‐	‐	‐	‐	‐	‐	‐	0.37	‐				
**IL12p70**	−0.33	‐	‐	‐	**0.52**	‐	‐	‐	‐	0.45			
**IP10**	‐	‐	‐	‐	‐	‐	‐	**0.57**	0.38	0.4	‐		
**TNFα**	‐	‐	‐	‐	‐	‐	‐	‐	‐	‐	‐	‐	
**IFIT3**	‐	‐	‐	‐	‐	‐	‐	‐	‐	0.79	‐	0.8	‐

^a^ Spearman Correlations (Chambers, 1998) with *p* < 0.001 shown in **Bold Type**, 0.001 ≤ *p* < 0.01 in Regular Type and 0.01 ≤ *p* < 0.05 in gray type, with *p *≥* *0.05 not shown. Dashes mark nonsignificant results. Gray boxes mark self‐correlations or duplicate conditions which are not shown. For all correlations, n = 40 (includes both SARS‐CoV‐2 positive and negative) except involving *IFIT3* mRNA or protein where n = 12.

^b^ IFIT3 protein was in units of log(fold‐change) after measurement by DIA.

### 3.10 Sensitivity testing

We performed sensitivity testing for all significant associations between IFN, inflammatory and ISG measurements with viral load (*N1* protein mRNA) by re‐examining the relationships after exclusion of patients without infection by SARS‐CoV‐2. In every case, we found similar relationships between each biomarker and viral load, increasing the confidence in our findings.

Because of the high degree of correlation between some biomarkers (Tables 11, 12, 13), for example, between *IFN*‐*β1* and *IFNα2* transcripts (Spearman correlation coefficient =1.00, *p* < 0.001), we examined the effect of adding a second biomarker as an adjustment to the relationship between each significant biomarker (*p* < 0.05) with the clinical diagnosis of infection (Tables 4, 5, 6, 8 and 9) or with viral load (Table 7 and individually reported results for *ACE2* mRNA, *IP*‐*10* mRNA and IP‐10 protein in text). In every case, we found similar results for the association between the biomarkers reported and infection status or viral load. A number of the biomarker measurements tested as adjustment variables appeared to have independent significant effects suggesting that significant and independent multivariable associations exist, however, our study is too small to report those results with confidence.

**TABLE 12:** Correlations between measured Proteins and mRNA biomarkers^a^.

mRNA, log(Fold‐Change)	Protein, log(pg/ml)^b^													
	GMCSF	IFNα2	IFNβ	IFNγ	IFNλ1	IFNλ23	IL1β	IL6	IL8	IL10	IL12p70	IP10	TNFα	IFIT3
***GMCSF***	‐	‐	‐	‐	‐	‐	‐	‐	‐	‐	‐	‐	‐	‐
***IFNα2***	‐	‐	‐	‐	0.32	‐	‐	‐	‐	‐	0.33	‐	‐	‐
***IFNβ1***	‐	‐	‐	‐	‐	‐	‐	‐	‐	‐	0.33	‐	‐	‐
***IFNγ***	‐	‐	‐	‐	‐	‐	‐	‐	‐	‐	‐	‐	‐	‐
***IFNλ1***	‐	‐	‐	‐	‐	‐	‐	‐	‐	‐	‐	0.49	‐	‐
***IFNλ2***	‐	‐	‐	‐	‐	‐	‐	‐	‐	‐	‐	0.36	‐	‐
***IFNλ3***	‐	‐	‐	‐	‐	‐	‐	‐	‐	‐	‐	‐	‐	‐
***IL6***	‐	‐	‐	‐	‐	‐	‐	‐	‐	‐	‐	−0.34	‐	‐
***IL8***	‐	‐	‐	‐	‐	‐	‐	‐	‐	‐	‐	‐	‐	‐
***IL10***	‐	−0.5	−0.44	−0.46	‐	**−0.65**	‐	‐	‐	‐	‐	‐	‐	‐
***IP10***	‐	‐	‐	−0.35	‐	−0.32	‐	0.45	‐	‐	‐	**0.61**	‐	‐
***TNFα***	‐	−0.37	‐	−0.33	‐	**−0.54**	‐	‐	‐	‐	‐	‐	‐	‐
***IFIT3***	‐	‐	‐	‐	‐	‐	‐	‐	‐	‐	‐	**0.87**	‐	0.73

^a^ Spearman Correlations (Chambers, 1998) with *p* < 0.001 shown in **Bold Type**, 0.001 ≤ *p* < 0.01 in Regular Type and 0.01 ≤ *p* < 0.05 in grey type, with *p *≥* *0.05 not shown. Dashes mark nonsignificant results. The only significant correlation between an mRNA and its protein is for IP‐10 which is moderately correlated but highly significant. For all correlations, n = 40 (includes both SARS‐CoV‐2 positive and negative) except involving *IFIT3* mRNA or protein where n = 12.

^b^ IFIT3 protein was in units of log(fold‐change) after measurement by DIA.

**TABLE 13:** Correlations among measured mRNA biomarkers^a^.

mRNA, log(Fold‐Change)	mRNA, log(Fold‐Change)											
	*GMCSF*	*IFNα2*	*IFNβ1*	*IFNγ*	*IFNλ1*	*IFNλ2*	*IFNλ3*	*IL6*	*IL8*	*IL10*	*IP10*	*TNFα*
***IFNα2***	‐											
***IFNβ1***	‐	**1.00**										
***IFNγ***	0.33	**0.52**	**0.5**									
***IFNλ1***	‐	**0.52**	**0.52**	0.45								
***IFNλ2***	‐	**0.53**	**0.52**	**0.59**	**0.86**							
***IFNλ3***	‐	0.44	0.44	0.47	**0.6**	**0.76**						
***IL6***	**0.55**	**0.6**	**0.59**	**0.45**	‐	0.34	**0.53**					
***IL8***	0.4	0.4	0.39	0.33	‐	‐	‐	‐				
***IL10***	0.48	‐	‐	0.4	‐	0.33	‐	‐	0.33			
***IP10***	‐	‐	‐	‐	0.34	0.36	‐	‐	**0.6**	0.5		
***TNFα***	0.43	‐	‐	‐	‐	‐	‐	‐	0.38	**0.77**	0.47	
***IFIT3***	‐	‐	‐	‐	‐	‐	‐	‐	‐	**0.67**	‐	‐

^a^ Spearman Correlations (Chambers, 1998) with *p* < 0.001 shown in **Bold Type**, 0.001 ≤ *p* < 0.01 in Regular Type and 0.01 ≤ *p* < 0.05 in gray type, with *p *≥* *0.05 not shown. Dashes mark nonsignificant results. Gray boxes mark self‐correlations or duplicate conditions which are not shown. For all correlations, n = 40 (includes both SARS‐CoV‐2 positive and negative) except involving *IFIT3* mRNA or protein where n = 12.

## 4 DISCUSSION

Our results show large increases in transcription of multiple genes involved in innate immune and inflammatory responses soon after SARS‐CoV‐2 infection and the development of viral‐like symptoms (Tables 4, 6, 7, and 9 and Figures 3, 4, 5). However, there was a broad‐based discrepancy in translation response relative to increased transcription signals similar to the host shut off patterns seen in multiple viruses, including human CoVs such as SARS‐CoV that have been reported by many and reviewed by others (Kamitani et al., 2009; Kikkert, 2020; Narayanan et al., 2008; Walsh et al., 2013; Xiao et al., 2008), and that is just beginning to be described in SARS‐CoV‐2 (Schubert et al., 2020; Thoms et al., 2020). An alternative possibility is that proteins are rapidly degraded after translation, however, either possibility is detrimental to a fully functional innate immune response.

Among the IFNs that we evaluated, several had large increases in transcription that were also strongly associated with viral load (Tables 6, 7), but protein production was either unchanged or decreased when comparing samples from symptomatic infected patients to controls with viral‐like symptoms not due to SARS‐CoV‐2 (Table 8). For proinflammatory cytokines, there were similar large increases in transcription (Table 4) but no change in measured protein production except for IP‐10 alone (Table 5). Considering that our samples were collected soon after initial symptoms from ambulatory patients, the protein production result may indicate that IP‐10 is among the first inflammatory proteins to increase early in infection.

The discrepancies between transcription and translation did not fully extend to the ISGs. We selected to evaluate these molecules because of their importance for antiviral defense (Blanco‐Melo et al., 2016; Diamond & Farzan, 2013; Haller et al., 2015; Hefti et al., 1999; Kochs & Haller, 1999; Kochs et al., 2002; Perreira et al., 2013; Sadler & Williams, 2008; Verhelst et al., 2013). Observed enormous increases in transcription (Table 9) were accompanied by several large increases in protein production (Table 10). Three antiviral ISGs had increased transcription and translation: *IFIT*‐*3* most strongly (Figure 5), *IFIT*‐*1* and *Tetherin*, and there was an additional borderline finding for *MX*‐*1* (Table 10). Of these ISGs, only *IFIT*‐*3* mRNA, however, was weakly correlated (*p* = 0.03) with its protein. These findings and the contrast in success of translation when compared with IFNs may be evidence of an IFN independent pathway for IFIT3 protein production (Bandyopadhyay et al., 1995; Liu et al., 2011).

In SARS, suppression of antiviral proteins occurred late in clinical disease (Cheung et al., 2005), however, our results suggest that with SARS‐CoV‐2, it occurs at the beginning of symptoms. Host translation suppression in SARS is associated with spike protein and nonstructural protein 1 (NSP1) interactions with eukaryotic initiation factor‐(eIF)‐3 which is required for protein translation (Xiao et al., 2008). Two recent publications investigating mechanisms involving Nsp1 for SARS‐CoV‐2 showed similar interference with eIF‐3 (Schubert et al., 2020; Thoms et al., 2020). Our results add to the *in vitro* work by demonstrating supportive evidence from early in the clinical course of human infection. Because viruses depend on host mechanisms for translation of viral proteins that are required for assembly of new infectious particles, our observation of continuing transcription and suppressed translation of human proteins may help explain persistent RT‐PCR detection of viral RNA but marked decreases in infectious viral particle production soon after the appearance of symptoms. These tentative hypotheses await further development and testing.

*ACE*‐*2* mRNA was increased in patients with SARS‐CoV‐2 infection. The finding indicates at least two possible causal relationships. SARS‐CoV‐2 may selectively infect people with existing high levels of *ACE*‐*2* transcription, or infection itself may increase transcription of *ACE*‐*2* above normal. In either case, increased transcription leading to increased protein expression of ACE‐2 likely would increase viral entry and thus help amplify viral replication.

We measured transcripts for three intracellular proteins important in pathways leading to IFN production and initiation of *NFκB*‐related inflammation. Transcription of *TBK*‐*1* and *STING*‐*1* were unchanged, whereas there was a decrease in *TRAF*‐*1* mRNA. TRAF‐1 is involved in several distinct inflammation‐related pathways, but a reduction is most likely associated with increased NFκB activity and subsequently increased systemic inflammation (Lalani et al., 2018). The other two proteins, TBK‐1 and STING‐1, are important for transmitting detection of viral invasion to processes that produce antiviral IFNs (Lei & Hilgenfeld, 2017). There was no increase in transcription of *STING*‐*1* and *TBK*‐*1*, however, *IFN*‐*λ1* and *IFN*‐*λ2* transcripts were markedly elevated (Table 6 and Figure 4). The increases in these transcripts were closely associated with viral load. These findings suggest that detection of viral invasion is successful in generating a signal to increase both systemic inflammation and IFN production. Massive and strongly significant increases in *IFN*‐*λ1* and *IFN*‐*λ2* mRNA (Table 6) may indicate the critical importance of Type III IFNs in SARS‐CoV‐2 (Andreakos & Tsiodras, 2020; Davidson et al., 2016; Jewell et al., 2010) even if protein production was decoupled from high levels of gene transcription at the time our samples were collected.

IP‐10 was the sole inflammatory cytokine detected with higher protein concentrations in our samples from infected patients (Table 5). IP‐10 promotes inflammation in Human Immunodeficiency Virus (Lei et al., 2019), H5 N1 Influenza A (Chan et al., 2005; Jong et al., 2006), Middle‐East Respiratory Syndrome virus (Chu et al., 2014) and SARS‐CoV (Chen & Subbarao, 2007) infections, thus its prominence early in SARS‐CoV‐2 infection, while unsurprising, may be important for understanding evolution of disease from initial mildly symptomatic to severe and sometimes fatal. Nevertheless, we found a moderately strong inverse relationship with age such that 10 or 25 additional years of age seemed to be associated with dampening of increases in IP‐10 (see Results). This inverse association is at odds with the clinical observation of worsening disease severity associated with older ages and generates questions about the nature of previously observed detrimental effects of IP‐10 (Huang et al., 2020; Lescure et al., 2020; Wang, Hu, et al., 2020; Zhou, Yang, et al., 2020) on morbidity and mortality with SARS‐CoV‐2 infection. Older individuals with COVID‐19, for example, may be more sensitive to suppression of antiviral defenses, whereas younger individuals are more sensitive to excessive inflammation. The observations and questions show that abnormal transcript and protein responses to infection cannot be fully interpreted without clinical context drawn from the evaluation of a larger study population.

In contrast to limited but interesting results with adjustments for age, adjustments for sex were uninformative. The lack of significant findings may be due to survivor biases. Ill and severely ill patients are less likely to be female (Huang et al., 2020), but the susceptibility to infection associated with sex remains unknown. Among patients who develop symptoms, innate immune responses may be similar regardless of sex.

Our study is limited by its cross‐sectional design, small size, and the nature of the nasopharyngeal swab samples. Due to the urgency of need, we obtained deidentified samples quickly in exchange for giving up detailed clinical annotation. We do not yet have sufficient information to interpret observed abnormalities in IFN and systemic inflammation to seek out associations with clinical outcomes such as respiratory failure or death. However, because a random sample of the population visiting our drive‐through diagnosis centers will contain predominantly survivors of infection who never require hospitalization, the measurements we report should roughly represent patients who generally suffer nonsevere disease.

The small size of the study limits our ability to generalize our interpretations and conclusions. Sensitivity analyses, however, increase our confidence in the stability of our findings and suggest that there are additional multivariable associations between biomarkers, infection status, and viral load that may be explored, further strengthening the impression that additional study of more individuals is needed to better understand the extent of innate immune disruptions due to SARS‐CoV‐2 infection.

Nasopharyngeal swab samples necessarily retrieve a variety of cell types and may retrieve secreted substances that originate elsewhere than the upper airway. Because samples were frozen prior to evaluation, characterization of cell types by cell counting or flow cytometry was not possible. A prospective human study with immediate processing to allow better assessments of cell types present is possible, but the full characterization of secreted molecules will require carefully designed cell culture models to prevent the inclusion of molecules produced elsewhere and transported to the nasopharynx. Two of the genes studied, *IFNα2* and *IFITM1* do not have introns, thus there is a small chance that our mRNA detection results could have been affected by amplification of genomic DNA despite examining RT‐PCR melt curves. However, the results of RT‐PCR showed that neither gene transcript was elevated thus our conclusions are unaffected. Finally, identification of other viruses causing presenting symptoms would provide insights on their transmissibility, however, because of small sample sizes, we did not assess for the presence of other viruses such as less pathogenic coronaviruses. More extensive nasopharyngeal sampling is noxious and reduces patient participation, thus future studies that target identification of other viruses may need to consider alternative methods of sampling or detection. Despite the limitations, however, our study provides information highlighting several areas of IFN and inflammatory biology that deserve future investigation.

Although our study identifies strong IFN and systemic inflammatory signal transcription responses to infection, only a larger prospective study incorporating careful annotation of patient characteristics, analysis of serial samples with disease progression and reporting of outcomes can fully assess the clinical implications of these initial findings. Our results overall, even with a small study size, emphasize that there are remarkable disruptions early in disease in the immune landscape. Further study is likely to be both fruitful and illuminating.

## CONFLICT OF INTEREST

TGL, JLS, and KAP are supported by NIH/NHLBI R01 125520 and by the US Cystic Fibrosis Foundation, Bethesda, MD, grants LIOU13A0, LIOU14P0, LIOU14Y4, LIOU15Y4. During the course of the study, TGL, JLS, and KAP received other support for performing cystic fibrosis‐related clinical trials from Abbvie, Inc; Corbus Pharmaceuticals Holdings, Inc; Gilead Sciences, Inc.; Laurent Pharmaceuticals, Inc; Nivalis Therapeutics, Inc; Novartis Pharmaceuticals; Proteostasis Therapeutics, Inc; Savara, Inc; TranslateBio; and Vertex Pharmaceuticals, Inc. None of the funding was provided for the development of vaccines or treatments for SARS‐CoV‐2 infection. FRA received other support from the National Science Foundation (grant EMSW21‐RTG) and NIH (NIH‐CSBC: U54 CA20). JEC is supported by NIH/NIDDK U54 DK110858 05. MNH is supported by NIH/NHLBI R01HL137033. CK received support from the UK Medical Research Council. DTL is supported by NIH/NAIAD R01AI130378. The sponsors of clinical trials and funders of other support played no roles in this study.

## AUTHOR CONTRIBUTIONS

TGL and FRA initiated the project. TGL, FRA, DRC, JEC, MNH, NDH, JLJ, CK, DTL, KAP, ABS, KJW, LJWa, LJWe, and RPIII designed the study. SCH oversaw the drive‐through tent clinical testing program. TGL, KEH, SCH, DTL, JLJ, SMS, and KT obtained samples. ABS and EZ performed RT‐PCR. GJG, MNH, YL, and JEM performed bead‐based multianalyte immunoassays. MNH, SMS, YL, and KT extracted RNA. JEC, KT, MNH, SMOS, and YL prepared proteins for mass spectrometry. JEC and SMOS performed mass spectrometry. TGL, FRA, DRC, JEC, and CK analyzed the data. TGL initiated the manuscript writing, but all authors, FRA, BCC, DRC, JEC, GJG, KEH, SCH, NDH, MNH, JLJ, CK, YL, DTL, JEM, EAM, SMOS, KAP, SMS, ABS, KT, KJW, LJWa, LJWe, EZ, and RPIII participated in writing, formulation of interpretations and in revisions. TGL, JEC, JEM, and RPIII obtained funding. JLJ, KAP, LJWa, and LJWe managed regulatory aspects of the study. Authors are listed in alphabetical order except first and senior (last) authors.
